# Identifying genetic variation associated with environmental gradients and drought‐tolerance phenotypes in ponderosa pine

**DOI:** 10.1002/ece3.10620

**Published:** 2023-10-13

**Authors:** Mengjun Shu, Emily V. Moran

**Affiliations:** ^1^ Life and Environmental Sciences University of California Merced California USA

**Keywords:** adaptive genetic variation, climate change, environmental association, GBS, phenotypic association, SNP

## Abstract

As climate changes, understanding the genetic basis of local adaptation in plants becomes an ever more pressing issue. Combining genotype‐environment association (GEA) with genotype–phenotype association (GPA) analysis has an exciting potential to uncover the genetic basis of environmental responses. We use these approaches to identify genetic variants linked to local adaptation to drought in *Pinus ponderosa*. Over 4 million Single Nucleotide Polymorphisms (SNPs) were identified using 223 individuals from across the Sierra Nevada of California. 927,740 (22.3%) SNPs were retained after filtering for proximity to genes and used in our association analyses. We found 1374 associated with five major climate variables, with the largest number (1151) associated with April 1st snowpack. We also conducted a greenhouse study with various drought‐tolerance traits measured in first‐year seedlings of a subset of the genotyped trees grown in the greenhouse. 796 SNPs were associated with control‐condition trait values, while 1149 were associated with responsiveness of these traits to drought. While no individual SNPs were associated with both the environmental variables and the measured traits, several annotated genes were associated with both, particularly those involved in cell wall formation, biotic and abiotic stress responses, and ubiquitination. However, the functions of many of the associated genes have not yet been determined due to the lack of gene annotation information for conifers. Future studies are needed to assess the developmental roles and ecological significance of these unknown genes.

## Graphical abstract

This study explores genetic variants and gene functions related to local drought adaptation in *Pinus ponderosa* (ponderosa pine) through combined genotype‐environment association (GEA) and genotype–phenotype association (GPA) analyses. Despite no individual SNP overlap between the analyses, the associated SNPs shared similar gene functional categories, such as the ubiquitination pathway, reproductive processes, cell wall formation, stress responses, and cell division and growth.

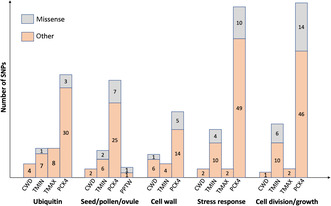

## 1 INTRODUCTION

Genomics promises exciting advances toward understanding adaptive genetic variation and evolutionary potential of plants under a rapidly changing and often increasingly variable environment (Capblancq et al., 2020; Harrisson et al., 2014; Hoffmann & Sgrò, 2011; Sang et al., 2022; Savolainen et al., 2013). Intraspecific genetic variation represents the potential for adaptive change in response to new selective challenges, which is critical for local species persistence under environmental change (Bell & Gonzalez, 2009; Brooker et al., 2022; Leites & Benito Garzón, 2023; Pauls et al., 2013; Rice & Emery, 2003). Adaptation to local climate conditions has been considered typical for tree populations (Kitzmiller, 2005; Langlet, 1971; Wright, 2007; Ying & Liang, 1994), but organisms with such long generation times and a sessile lifestyle can become maladapted if environmental shifts rapidly occur (Aitken et al., 2008; Alberto et al., 2013; Benomar et al., 2022; Frank et al., 2017; Gougherty et al., 2021). Plants also exhibit plastic changes in their growth form and physiology in response to stress, and the level of plasticity can itself be heritable (Auld et al., 2010; de la Mata et al., 2022; Van Kleunen & Fischer, 2005; Wu et al., 2023; Zeng et al., 2017) and may be under the selection (Zettlemoyer & Peterson, 2021). Understanding the distribution of genetic variation related to environmental responses may help us better predict changes and manage forests in a shifting climate (Leites & Benito Garzón, 2023; Neale & Kremer, 2011; Oney et al., 2013; Razgour et al., 2019). This includes selecting seed sources for restoration or breeding that have desirable characteristics such as drought tolerance (Beaulieu et al., 2014; Cortés et al., 2020; Isik, 2014; Ray et al., 2022).

Landscape genomics offers enormous potential to discover genes responsible for local adaptation by investigating the statistical association between genetic variation at individual loci and the putative causative environmental factors (Eckert et al., 2010, 2015; Feng & Du, 2022; Lu et al., 2019; Shaffer et al., 2022; Sork et al., 2013). This approach is sometimes known as genotype‐environment association (GEA) analysis. Prior studies in *Arabidopsis* – the primary plant model organism – have found that environmentally associated SNPs can predict performance in common gardens (Hancock et al., 2011). A *Pinus pinaster* study suggests this could be true in trees as well, even when only a modest number of the genetic variants involved have been identified (Jaramillo‐Correa et al., 2015). However, GEA studies do not by themselves reveal why specific alleles are more prevalent in particular environments – for example, are they responsible for selectively favored traits? Genotype–phenotype association (GPA) analysis identifies loci linked to a specific phenotype (Depardieu et al., 2021; Eckert et al., 2009; Holliday et al., 2010; Housset et al., 2018; Santini et al., 2021). In plant GPA studies, individuals are typically grown in a common environment to eliminate the effects of environmental variation on phenotypes. However, this approach does not reveal whether a trait variant would be favored in the field. GEA and GPA association are thus complementary, and combining them might better identify the loci and traits that are selectively favored in particular conditions than either could alone (Eckert et al., 2015; Mahony et al., 2020; Talbot et al., 2017).

The large genome size of conifer trees (>19 GBP) represents a challenge for analysis. Most association studies in conifers have focused on Single Nucleotide Polymorphisms (SNPs) within a few hundred genes (Dillon et al., 2014; Eckert et al., 2009, 2015; Hamilton et al., 2013; Holliday et al., 2010; Housset et al., 2018), or fewer than 2000 genome‐wide SNPs (Uchiyama et al., 2013). One notable exception is a recent study on lodgepole pine that used a sequence capture dataset created by mapping the *Pinus contorta* transcriptome to the *Pinus taeda* genome sequence (Mahony et al., 2020). Most genome‐wide studies, however, are limited to pines species with a full genome sequence (Cappa et al., 2022; De La Torre et al., 2019; Lu et al., 2019; Weiss et al., 2022). Still, most conifers have neither a published genome sequence nor a complete transcriptome. Though targeted sequencing is efficient, candidate gene approaches may miss other vital genes with previously unsuspected roles in local adaptation, and focusing solely on variants within genes may miss significant variants within regulatory regions.

Several approaches to identifying more genetic variants for genome‐wide association studies (GWAS) utilizing next‐generation sequencing (NGS) have been proposed in recent years (Badenes et al., 2016; Davey et al., 2011; Poland & Rife, 2012; Younessi‐Hamzekhanlu & Gailing, 2022). Genotyping‐by‐Sequencing (GBS), which can generate tens of thousands of SNP markers without the need for a reference genome or whole transcriptome, has emerged as a cost‐effective strategy (Andrews et al., 2016; Elshire et al., 2011). By combining the power of multiplexed NGS with restriction‐enzyme‐based genome complexity reduction, GBS enables the genotyping of large populations for thousands of SNPs in an increasingly rapid and inexpensive way (Poland et al., 2012; Poland & Rife, 2012).

Despite the high economic and ecological importance of ponderosa pine (*Pinus ponderosa*) in the western United States (Graham & Jain, 2005), no previous study has attempted to identify the relationship between gene sequence variation and drought tolerance in this species. Some studies have investigated the evolutionary history and phylogeography of *P. ponderosa* using mitochondrial DNA markers; these reflect the long‐term biogeographical process contributing to the modern distribution of the species but have limited adaptive significance in themselves (Johansen & Latta, 2003; Potter et al., 2013). Other studies have emphasized the importance of intraspecific variation of *P. ponderosa* in environmental responses but focus on the phenotypic variation within and among populations without identifying the underlying genetic variation (Kolb et al., 2016; Maguire et al., 2018). California's historic 2012–2016 drought may represent an increasingly common condition as climate changes (Berg & Hall, 2015; Griffin & Anchukaitis, 2014). Such “hot droughts” can lead to mass tree mortality, even in relatively drought‐tolerant species like ponderosa pine, negatively impacting the sustainability of conifer forests (Fettig et al., 2019). A deep understanding of the genetic basis of adaptation in ponderosa pine and other conifers in the western United States is critical for successful reforestation and conservation programs.

In this study, we delve into the genetic basis of local adaptation and drought‐response traits in ponderosa pine populations from diverse climates within the central Sierra Nevada mountains in California. Using a comprehensive approach, we performed a GEA analysis on 223 genotypes and, subsequently, conducted a GPA analysis on seedlings germinated from a selected subset of these trees. We also made use of gene annotation to assign biological functions to genes linked with or adjacent to the identified SNPs. The aims of the present study were to unravel the genetic underpinnings of climate adaptation and drought‐responsiveness in ponderosa pines through combined GEA and GPA analyses and (2) to integrate association studies with gene annotation analysis to spotlight genes and functions of significance for adaptation. We hypothesized that certain gene functions previously identified as important for drought tolerance in trees (Moran et al., 2017) – such as those in the abscisic acid (ABA) signaling pathway used to close the stomata during stress – would be identified in both analyses, but that new functions would be identified as well.

## 2 MATERIALS AND METHODS

### 2.1 Sampling and DNA sequencing

**FIGURE 1:**
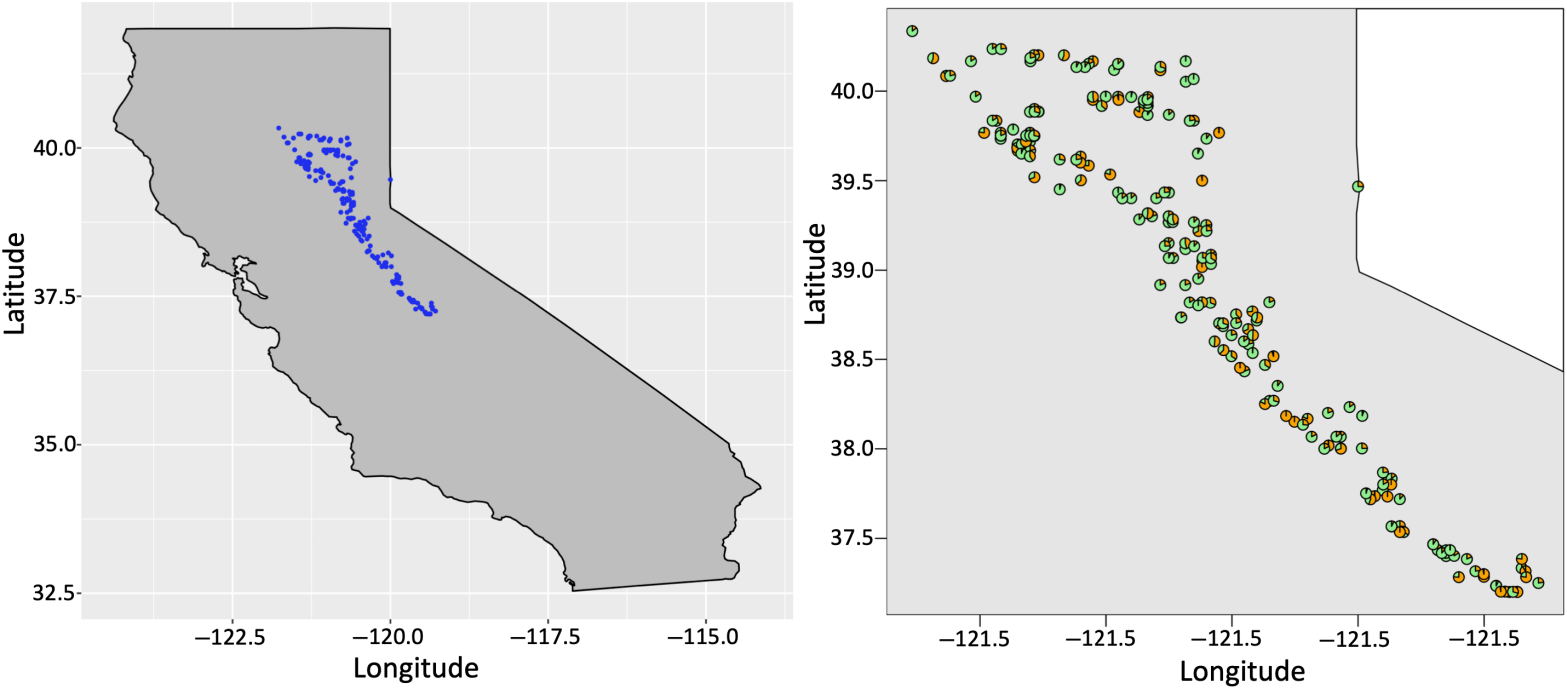
Source location and the admixture analysis of the 223 ponderosa pine genotypes. Left: Original geographic distribution of the 223 ponderosa pine genotypes. Right: Proportion of each individual's genome allocated to “population 1” (green) and “population 2” (orange) by admixture analysis when *K* = 2, illustrating lack of geographical structure. Trees were subsequently treated as part of a single population.

In the 1970s, the Forest Service's Pacific Southwest Regional Genetic Resources Program planted clones of 302 wild ponderosa pines from diverse climate conditions in the central portion of the Sierra Nevada mountains in an orchard located in Chico, California. From this orchard, we selected 223 individual *P. ponderosa* genotypes for the GEA analysis, ensuring they spanned the full climatic range represented in the original collection (Figure S1). The source locations for these genotypes (Figure 1) fell within just one of the several genetic subdivisions previously identified in ponderosa pine (Conkle & Critchfield, 1988; Potter et al., 2015; Williams, 2009). Fresh needles were collected from these individuals and placed in labeled tea bags over silica gel to dry them and quickly preserve the DNA for extraction.

DNA was extracted from the dried needles using a modified Qiagen plant kits protocol by adding proteinase K and quantified using an Eppendorf BioSpectrometer (Eppendorf, AG, Germany). Samples were frozen and sent to the UC Davis Genome Center for library construction. Four 48‐plex GBS libraries consisting of 47 DNA samples and negative control (no DNA) and one 36‐plex GBS library composed of 35 DNA samples and negative control were prepared. The pool was quantified via qPCR using the KAPA Library Quantification Kit (Kapa Biosystems, Wilmington, MA, USA) for Illumina sequencing platforms, with 0.9X bead cleanup to remove small fragments (<250 bp). Additional DNA purification using the Zymo DNA Clean & Concentrator kit (Zymo Research, Irvine, CA) was performed to increase the purity of the extracted DNA. The libraries were then sequenced (single‐end read 90 or 100 bp) using an Illumina HiSeq 4000 (Illumina, San Diego, CA), one library per lane.

### 2.2 SNP calling and filtering

No reference genome is available for ponderosa pine (*P. ponderosa*), but one does exist for loblolly pine (*P. taeda*) (Neale et al., 2014; Zimin et al., 2014, 2017). Of the conifers that have been sequenced to date, *P. taeda* is the most closely related to *P. ponderosa* (Gernandt et al., 2009; Willyard et al., 2009). Furthermore, the *P. taeda* reference genome was successfully used to design probes for sequence capture in *P. contorta* (Suren et al., 2016; Yeaman et al., 2016), a distant relative. Based on preliminary analyses, we selected the Stacks v.2.2 pipeline (Rochette & Catchen, 2017) with this reference genome (https://treegenesdb.org/FTP/Genomes/Pita/) for SNP calling (Shu, 2020). Each step in the Stacks reference pipeline was performed internally in Stacks algorithms except alignment with BWA v.0.7.17 (Li & Durbin, 2009) and the Samtools v.1.9 (Li, 2011) step used to get read position. Default settings were used in Stacks, BWA, and Samtools.

After calling the SNPs, we ran SnpEff (Cingolani et al., 2012) to identify the location of the gene containing each SNP. We used the database of annotated genome and the reference genome of loblolly pine v.2.01 in TreeGenes (http://treegenesdb.org/FTP/Genomes/Pita/v2.01/). The location of each SNP was listed in the output file of SnpEff as one of six primary location categories, including intragenic variants, intergenic variants, upstream SNPs, downstream SNPs, synonymous, and missense variants in the gene coding sequence. In SnpEff, “intragenic” refers to SNPs in introns, while “missense” refers to any non‐synonymous mutation in the transcribed region.

Many SNPs identified by GBS fell between genes and regulatory regions (in the intergenic regions) and likely had no direct effect on gene expression or function. In addition, because of the low amount of linkage disequilibrium in conifers (Isik et al., 2016; Namroud et al., 2008), any associations identified between such intergenic SNPs and a phenotype or environment of interest were likely false positives rather than reflecting linkage between the SNP and a causal variant. Therefore, we first filtered out the intergenic SNPs before running the association analysis using a Python script (https://github.com/shumengjun/LFMM).

### 2.3 Climate data

We obtained 30‐year (1921–1950) averages of climate data for each genotype source location from the 270 m resolution California Basin Characterization Model (BCM) (Flint et al., 2013). These mid‐20th‐century values were used instead of more recent climate data because they more closely resemble the conditions when the genotypes were establishing as seedlings. For the GEA analysis, a PCA was conducted on the entire climate dataset to determine key climatic variables. The first two principal components captured a significant 68.2% of the total climatic variation (Figure S2). We decided to focus our analysis on five crucial climate variables components that contributed strongly to the first two principal components, including: mean climatic water deficit (CWD, a measure of evaporative demand exceeding soil moisture); mean minimum winter (December–February) temperature (TMIN); mean maximum summer (June–August) temperature of summer (TMAX); mean monthly winter precipitation (PPTW); and mean April 1st snowpack (PCK4). Other climate variables considered but not included in the analysis were actual evapotranspiration (AET), potential evapotranspiration (PET), mean monthly summer precipitation (PPTS), excess water (EXC), recharge (RCH), runoff (RUN), snowfall (SNW), snowmelt (MLT), soil water storage (STR), and snow sublimation (SBL).

### 2.4 Genotype‐environment association analysis

We used latent factor mixed model 2 (LFMM2) for GEA association, which has been shown to outperform similar approaches with several orders‐of‐magnitude faster computing (Caye et al., 2019), which also controls for the effects of demographic processes and population structure (Wang et al., 2017). This approach is robust to high amounts of missing data, such as GBS sequencing tends to produce, when sample sizes are >100 (Xuereb et al., 2017).

LFMM2 regression models combine fixed and latent effects with the following equation:

$$Y={\mathrm{XB}}^{T}+W+E.$$

**Y** is a matrix of genetic information measured from *p* genetic markers for *n* individuals, and **X** is a matrix of *d* environmental variables measured for *n* individuals. The fixed effect sizes are recorded in the **B** matrix, which has dimension *p* × *d*. The **E** matrix represents residual errors with the same dimensions as the response matrix. The matrix **W** is a matrix of rank *K*, defined by *K* latent factors where model choice procedures can determine *K*. The *K* factors represent unobserved confounders – usually geographical structure in the genotypes of the samples – defined as an *n* × *K* matrix, **U**. **V** is a *p* × *K* matrix of loadings. The matrix **U** is obtained from the matrix's singular value decomposition (SVD):

$$W={\mathrm{UV}}^{T}.$$

We used the two approaches implemented in the LEA v.2.6.0 R package to determine *K*: principal component analysis (PCA) and admixture analysis (Frichot et al., 2013; Frichot & François, 2015). First, we ran the LEA function PCA to select the number of significant PCA components by computing Tracy‐Widom tests with the LEA function Tracy.widom (Patterson et al., 2006). Second, we ran the LEA function snmf for *K* values between 1 and 5 with 10 repetitions each. The most likely *K* value was identified by minimizing the cross‐validation error evaluated in the 10‐fold cross‐validation procedure. Upon executing the GEA using LFMM2 with the determined *K* value, we calibrated the raw *p* values by employing the Genomic Inflation Factor (GIF) to account for potential distortions caused by population structure or other intervening variables. We then chose significant associations based on *p* (<10^−5^) value. This calibration, combined with our threshold criteria, was pivotal in ensuring stringent False Discovery Rate (FDR) control, affirming the credibility of our identified associations.

### 2.5 Greenhouse experiment and phenotype measurements

In this study, we conducted a greenhouse experiment with both wet and drought treatments in order to carry out the GPA. The specific procedures for the greenhouse experiment and the phenotype assessments are described in Wu et al. (2023). We selected 50 seed sources among our 223 genotypes that still represent the same breadth of climate conditions as the full set of trees (Figure S3); greenhouse size did not allow for a larger sample of families. We aimed to have 10 seedlings from each maternal family in both wet and dry treatments, 1000 seedlings in total. As responses to dry versus wet conditions could not be measured in the genotyped adult individuals, we used average values for their offspring.

We recorded nine seedling traits: height growth (GR; in centimeters), root length (RL; in centimeters), dry shoot weight (SW; in grams), dry root weight (RW; in grams), the ratio of root‐to‐shoot dry mass (R2S), specific root length (SRL; in centimeters per gram), stomata density of adaxial side (SD_AD; in numbers per square millimeter), the number of stomatal rows on the abaxial side (NR_AB; in number per mm^2^), and the number of stomatal rows on the adaxial side (NR_AD; in number per mm^2^). Forty‐two maternal families had sufficient germination to enable these measurements across both wet and drought treatments.

### 2.6 Genotype–phenotype association analysis

We used the SNPs identified in the 42 mother trees for the GPA association analysis, focusing on the traits significantly associated with drought treatments. Two groups of traits' measurements were included in the GPA analysis. For the control treatment traits, we used the average trait value across all members of each family in the wet treatment to run GPA analysis. For the drought responsiveness, we deducted the average trait value for a given family in the wet treatment from the value for each family's offspring in the drought treatment. We used LFMM 2 (Caye et al., 2019) for GPA analysis, using trait measurements as explanatory variables, in contrast to the environmental variables used in the GEA analysis, with the explanatory variables as the traits' measurement instead of environmental variables in GEA analysis. Following this, we calibrated the raw *p* values from the GPA analysis using the GIF to correct for potential biases introduced by population structure or other confounding factors. Associations were deemed significant based on *p* (<10^−5^) value. This calibration approach, along with our chosen threshold, was instrumental in ensuring rigorous control of the FDR, thereby enhancing the reliability of our GPA analysis results.

### 2.7 Gene annotation

After identifying the significantly associated SNPs in GEA and GPA, we aligned the gene sequences for these regions against the nonredundant protein sequences database using UniProt to identify the gene and protein with the implemented Blastx (2.9.0+, *E* < 1e^−10^). The Gene Ontology Annotation Database (Bateman et al., 2017; UniProt Consortium, 2015) was used to identify the potential functions of the genes further. If a SNP is in the intragenic region, we performed a search by querying the flanking sequence 400 bp from the beginning position of the gene. This step was essential because, for genes encompassing introns, the distance between the “start” and “end” positions was considerable, often resulting in Blastx yielding no matches.

## 3 RESULTS

### 3.1 Genetic diversity and population structure

A total of 4,155,896 SNPs were identified from GBS data of the 223 genotypes after initial filtering. With these SNPs, we ran both principal component analysis (PCA) and admixture analysis to determine the number of populations (*K*) represented by these individuals. Remarkably, the PCA indicated that all 223 genotypes clustered closely together, as depicted in Figure S4. Despite the broad geographical range of our samples, they appear to represent a single population. This observation is consistent with previous research, which posits that the ponderosa pines in the Sierra Nevada mountains belong to one of the previously identified genetic subdivisions (Potter et al., 2015). Even though our samples are across a wide distribution, it belongs to the same population, which is also in accordance with the previous findings, which indicate the ponderosa pine in Sierra Nevada mountains belongs to one of the previously identified subdivisions. According to the admixture analysis result, the best *K* value was one (Figure S5). We also plotted the admixture of each individual tree. We found that the identified “populations” when *K* = 2 completely overlapped geographically (Figure 1b, Figure S6). Thus, we concluded that the sampled genotypes belong to one interbreeding population and used *K* = 1 for the association analysis.

### 3.2 Environmental associations at individual loci

**FIGURE 2:**
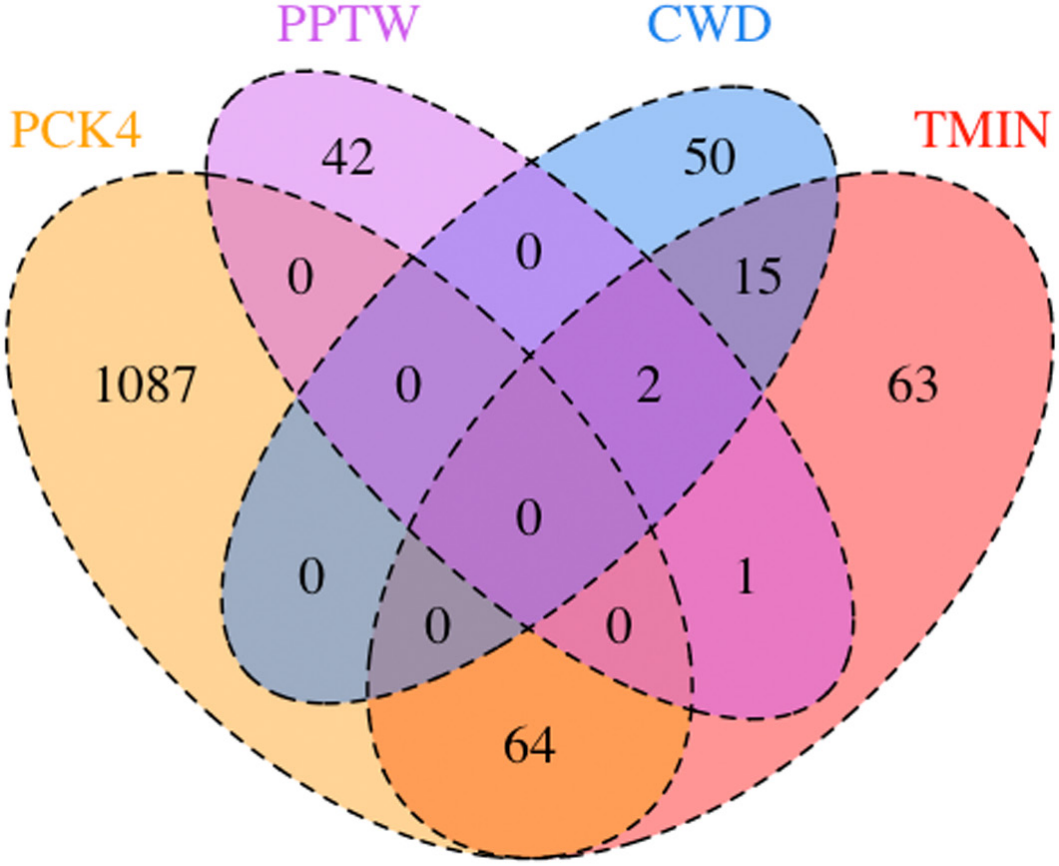
Venn diagram comparing overlap in environmentally associated SNPs. The number of overlapping SNPs that are associated with four climatic variables between April 1st snowpack (PCK4), monthly winter precipitation (PPTW), climatic water deficit (CWD), and minimum winter temperature (TMIN).

After filtering out the intergenic SNPs that might result in false positives, we were left with 927,740 (22.3%) SNPs in or near genes. These were then used for the association analyses. After the running of LFMM2 (*p* < 10^−5^) for GEA, we found 1374 significant associations with the five selected environmental variables (Table 1). PCK4 (April 1st snowpack) had the most associations, with TMIN (minimum winter temperature) having the following highest number. Few SNPs were associated with more than one climatic variable, with the highest degree of overlap between PCK4 and TMIN (64 SNPs) and between CWD and TMIN (17 SNPs) (Figure 2).

**TABLE 1:** Number of environmentally associated SNPs located in different regions.

Location of SNP	PCK4	TMIN	CWD	TMAX	PPTW
Upstream	335 (29%)	33 (23%)	11 (16%)	12 (24%)	16 (36%)
Intragenic (intron)	336 (29%)	34 (23%)	24 (36%)	18 (36%)	7 (16%)
Synonymous	92 (8%)	22 (15%)	6 (9%)	5 (10%)	2 (4%)
Missense	157 (14%)	20 (14%)	2 (3%)	11 (22%)	5 (11%)
Downstream	229 (20%)	36 (25%)	24 (36%)	3 (6%)	15 (33%)
Other	2 (0.1%)	0	0	1 (2%)	0
Total	1151	145	67	50	45

For PCK4 and TMIN, there were roughly similar numbers of associated SNPs in upstream and downstream regions versus the gene itself, with 14% of associated SNPs being missense (non‐synonymous) mutations (Table 1). SNPs associated with CWD were also roughly evenly split between flanking regions and the main gene sequence, but only 3% were missense mutations. A higher proportion of SNPs associated with TMAX (maximum summer temperature) were within the gene (68%), with 22% being missense mutations, while PPTW (winter precipitation) showed the opposite pattern, with 69% of SNPs being in the flanking regions.

### 3.3 Phenotypic associations at individual loci

Although 50 maternal families were initially selected for the greenhouse experiment, only 42 had sufficient germination for measurements to be included in analyses. Six out of the eight measured phenotypic traits were significantly different in the drought treatment versus the wet treatment. GR and SW decreased, while RL, R2S, SD_AD, and NR_AB increased. We therefore focused on these traits for the following GPA analysis, including both the average measurement of control treatment family and drought responsiveness for each trait. Heritabilities of trait responses to drought ranged from 0.15 to 0.65, and are discussed further in Wu et al. (2023), with variation in shoot growth in response to drought having the highest heritability.

**FIGURE 3:**
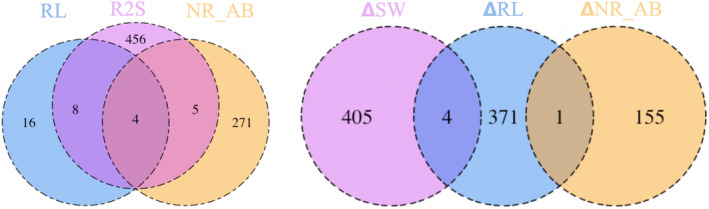
Venn diagram comparing overlap in phenotypically associated SNPs. Left: Overlap in SNPs significantly associated with control root length (RL), root‐to‐shoot ratio (R2S), and abaxial stomatal rows (NR_AB). SNPs associated with control height growth (15), adaxial stomatal density (12), and shoot weight (9) did not overlap with other categories. Right: Overlap in SNPs significantly associated with drought responsiveness of shoot weight (ΔSW); root length (ΔRL); and the number of stomatal rows on abaxial side (ΔNR_AB). SNPs associated with drought responsiveness of height growth (150), adaxial stomatal density (57), and R2S (6) did not overlap with any other categories.

More SNPs were associated with the trait drought responses (1149) than with the control traits (796). While control R2S had the most associations and SW the least (Table 2), the opposite was the case for drought responsiveness (Table 3). The number of SNPs associated with more than one trait was low in both GPA analyses. The highest degree of overlap was in control traits of RL and R2S (12 SNPs) and of R2S and NR_AB (nine SNPs) (Figure 3). The proportion of associated upstream SNPs was similar across control traits (32%–43%), but proportions of other categories varied widely, with the proportion of missense SNPs ranging from 8% to 25% (Table 2). For drought response, the distribution of SNPs in all categories differed, with the proportion of upstream being 19%–34% and the proportion of missense being 7%–16% for traits other than R2S (Table 3). R2S was only associated with six SNPs, five upstream and one downstream.

**TABLE 2:** Number of SNPs associated with traits in control conditions.

Location of SNP	R2S	NR_AB	RL	GR	SD_AD	SW
Upstream	166 (35%)	90 (32%)	12 (43%)	6 (40%)	4 (33%)	3 (33%)
Intragenic (intron)	106 (23%)	79 (28%)	5 (18%)	2 (13%)	3 (25%)	1 (11%)
Synonymous	40 (8%)	18 (6%)	1 (3%)	0 (0%)	2 (17%)	1 (11%)
Missense	61 (13%)	21 (8%)	3 (11%)	3 (20%)	3 (25%)	2 (22%)
Downstream	100 (21%)	72 (26%)	7 (25%)	4 (27%)	0 (0%)	1 (11%)
Other	0 (0%)	0 (0%)	0 (0%)	0 (0%)	0 (0%)	1 (11%)
Total	473	280	28	15	12	9

**TABLE 3:** Number of SNPs associated with drought responsiveness of traits.

Location of SNP	ΔR2S	ΔNR_AB	ΔRL	ΔGR	ΔSD_AD	ΔSW
Upstream	5 (83%)	43 (28%)	84 (22%)	48 (33%)	11 (19%)	138 (34%)
Intragenic (intron)	0 (0%)	41 (26%)	115 (30%)	41 (27%)	33 (58%)	113 (28%)
Synonymous	0 (0%)	10 (6%)	29 (8%)	11 (7%)	1 (2%)	43 (10%)
Missense	0 (0%)	15 (10%)	60 (16%)	15 (10%)	4 (7%)	46 (11%)
Downstream	1 (17%)	45 (29%)	85 (23%)	35 (23%)	8 (14%)	69 (17%)
Other	0 (0%)	2 (1%)	3 (1%)	0 (0%)	0 (0%)	0 (0%)
Total	6	156	376	150	57	409

### 3.4 Gene annotation for the significantly associated SNPs

**FIGURE 4:**
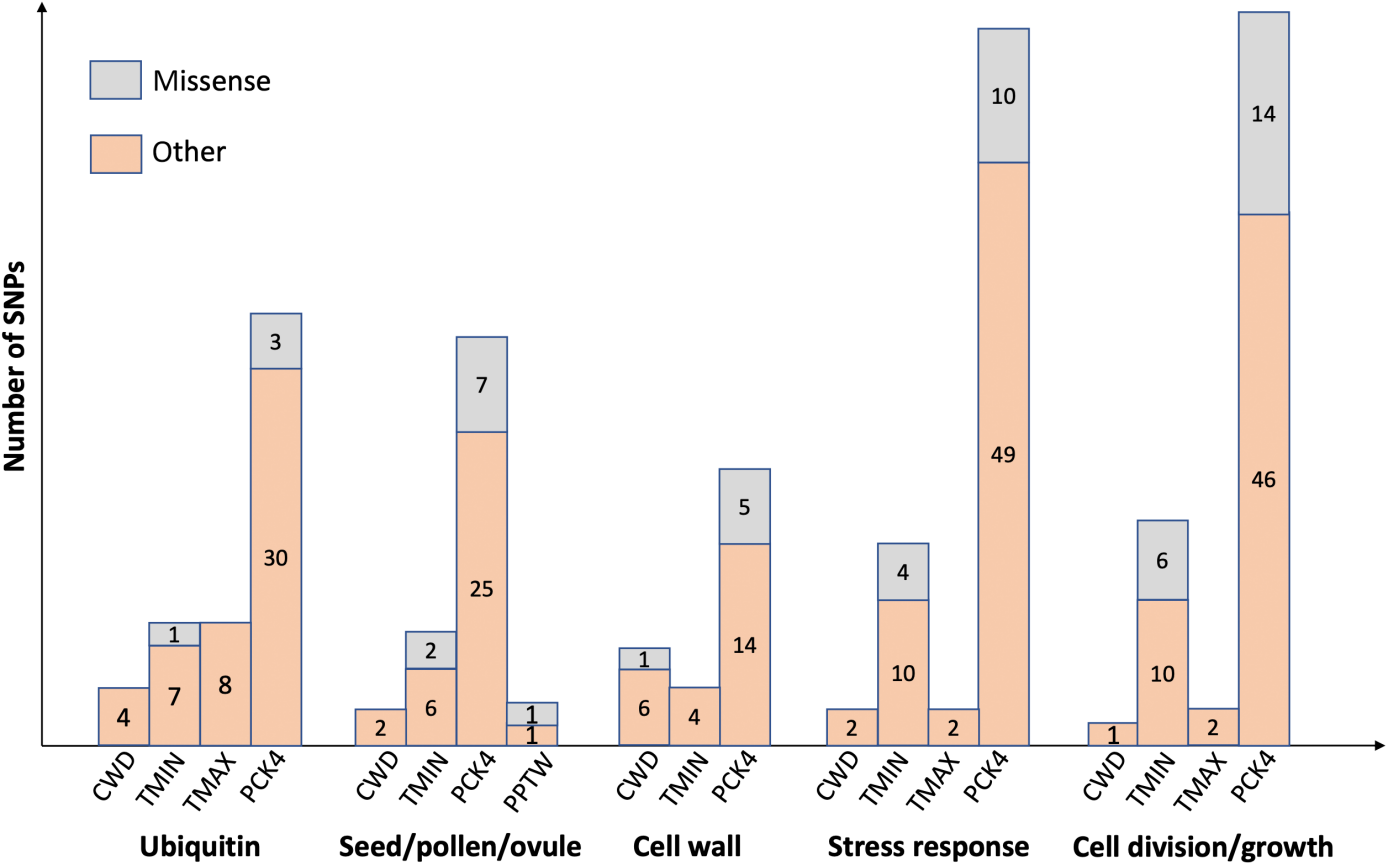
Five types of annotated SNP functions associated with different climatic variables. The number of non‐synonymous variants and other variants that are associated with the five climatic variables: Climatic water deficit (CWD); Minimum winter temperature (TMIN); Maximum summer temperature (TMAX); April 1st snowpack (PCK4); and Monthly winter precipitation (PPTW). Missense (non‐synonymous) SNPs are shown in gray, and other types of SNP are in orange.

Of the 1374 SNPs associated with environmental gradients, functions could be assigned for 788 (54%), while the rest had no matches in available gene ontology databases. We found that 283 SNPs with identifiable functions belonged to protein types that may be directly related to drought tolerance or other environmental responses (Figure 4). We categorized these genes into five main functional groups: (a) the ubiquitination pathway, (b) seed, pollen, and ovule formation, (c) cell wall formation, (d) stress responses, and (e) cell division and growth. Other associated SNPs with known functions were in or near transcription factors and genes with expression‐regulating functions.

Many of the SNPs associated with TMAX, TMIN, CWD, and PCK4 were in or near genes in the protein ubiquitination pathway or the jasmonic acid synthesis response pathways (Figure 4), both of which are involved in responses to biotic or abiotic stress (Creelman & Mullet, 1995; Lyzenga & Stone, 2012; Stone, 2014). CWD and PCK4 were also associated with SNPs in or near genes involved in seed dormancy, cell wall organization, and the abscisic acid (ABA) signaling pathway, which have been previously linked to drought responses in trees (Moran et al., 2017). Genes involved in reproduction, including pollen and ovule formation, were associated with TMAX, TMIN, and PCK4. Genes involved in vascular tissue formation, growth regulation, and stress responses were associated with TMAX and PCK4. Genes involved in stomatal regulation and pathogen responses were associated with TMIN and PCK4. Further biotic and abiotic stress response genes were associated with PCK4, as were genes involved in nutrient transport, photosynthesis, respiration, sugar synthesis, and light responses.

**FIGURE 5:**
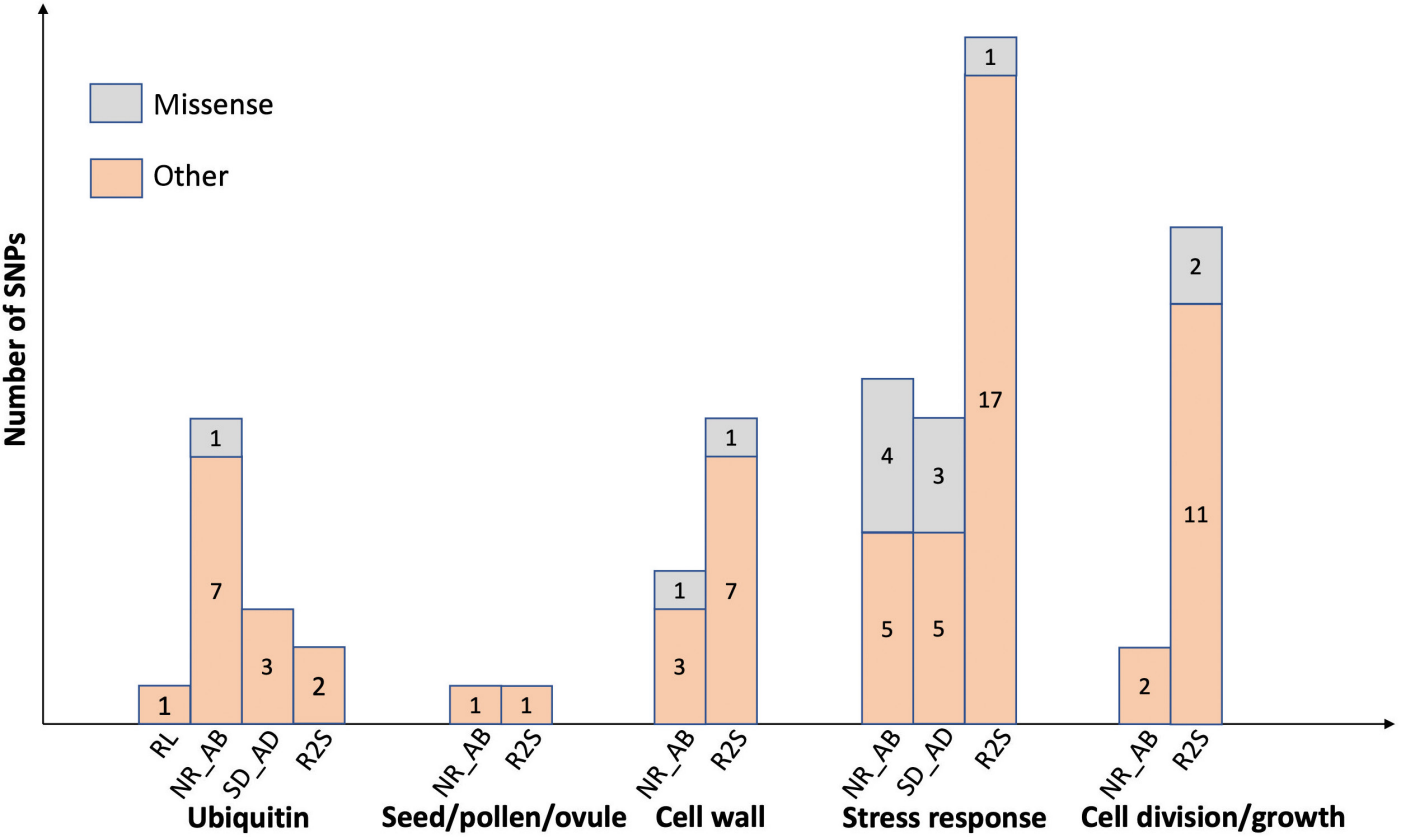
Five types of annotated SNP functions associated with different traits in control conditions. The number of non‐synonymous variants and other variants that are associated with four traits in control conditions: root length (RL), number of stomatal rows on abaxial surface (NR_AB), stomatal density on adaxial surface (SD_AD), and the ratio of root‐to‐shoot dry mass (R2S). No SNPs in these categories were associated with height growth or shoot weight. Missense (non‐synonymous) SNPs are shown in gray, and other types of SNP are in orange.

**FIGURE 6:**
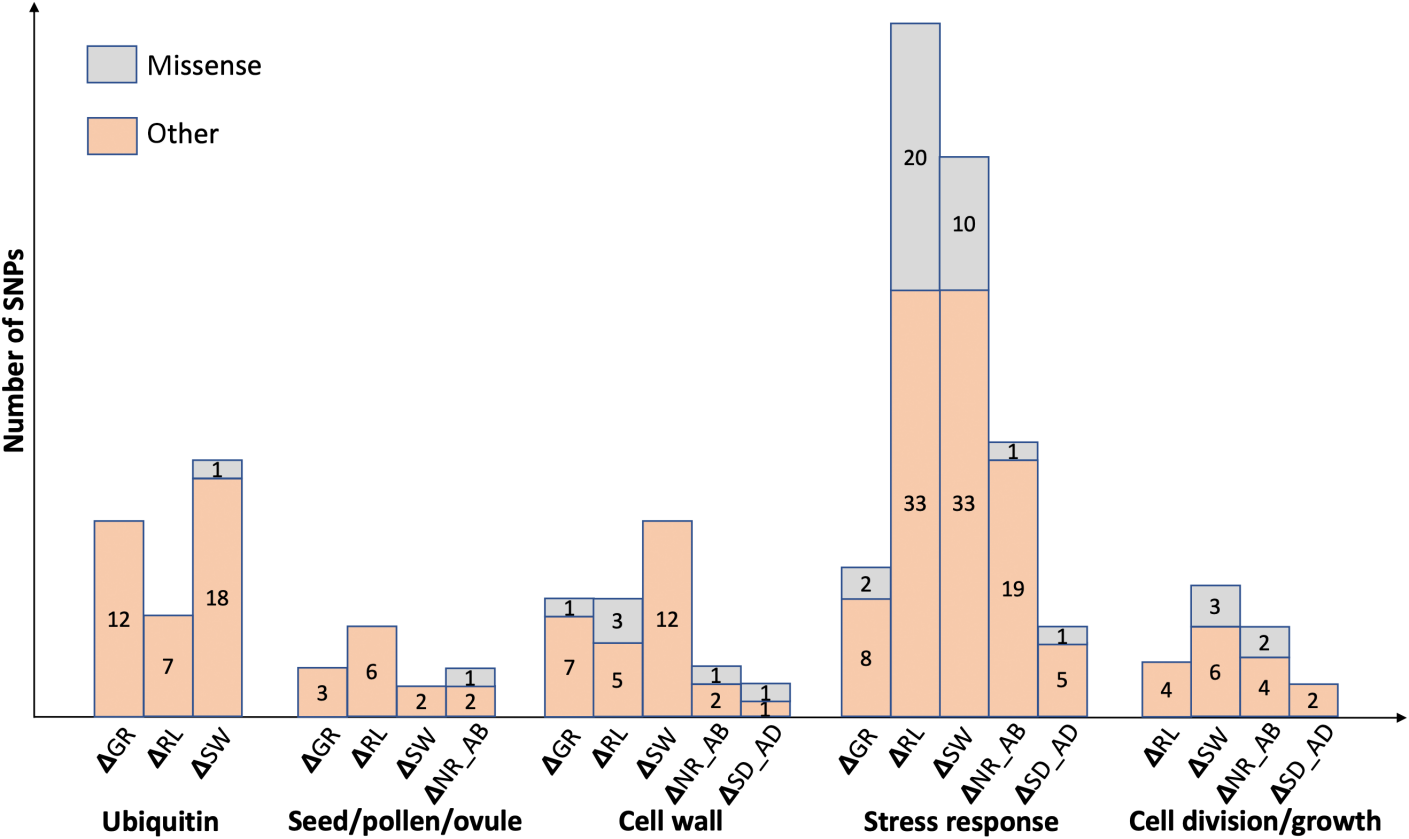
Five types of annotated SNP functions associated with drought responsiveness of different traits. The number of non‐synonymous variants and other variants that are associated with drought responsiveness of five traits: changes in height growth (GR), root length (RL), dry shoot weight (SW), number of stomatal rows on abaxial surface (NR_AB), and stomatal density on adaxial surface (SD_AD). No SNPs in these categories were associated with the ratio of root‐to‐shoot dry mass (R2S). Missense (non‐synonymous) SNPs are shown in gray, and other types of SNP are in orange.

Of the 796 SNPs associated with seedling control (wet treatment) trait values and 1149 SNPs associated with trait drought responsiveness, 43% and 51% could be assigned functions by gene ontology. Many of the same functional categories of genes associated with the environment were also related to measured phenotypes. This includes ubiquitination, seed development, cell wall organization, stress response, cell division (Figures 4, 5, 6), and transcription factors. However, there was no overlap in specific SNPs identified in control and drought responsiveness traits.

The control treatment levels of the two stomatal traits (NR_AB and SD_AD) were associated with genes involved in ubiquitination, cell wall organization or modification, growth and development, and ABA response. Control R2S was associated with genes involved in biotic & abiotic stress responses, cell wall organization or modification, cell division or differentiation, lateral root formation, and ubiquitination. Control height growth had no associated SNPs, and root length was only associated with one SNP located in a gene involved in ubiquitination (Figure 5). However, drought responsiveness of height growth, shoot weight, and root length was associated with all five functional categories (Figure 6). Drought responsiveness of the two stomatal traits was associated with genes involved in stress responses, cell wall formation/organization, cell division/differentiation, and root formation.

Besides the five main functional groups of genes with SNPs associated with climatic, phenotypic, and drought response variables, several other functional groups were identified in the GEA and GPA annotation results. For example, 111 (14%) of the environmentally associated SNPs, 53 (6%) of SNPs associated with control traits, and 121 (12%) of the SNPs associated with trait drought responses were in genes relating to ATP binding or protein kinases. It was also fairly common for associated SNPs to be in genes associated with RNA/DNA binding, metal ion binding, translation, and protein transport.

### 3.5 Overlapping annotated genes in GEA and GPA

While, as noted in the section above, there was no overlap in the exact SNPs identified by GEA and GPA analyses, a few of the associated SNPs were found to be in the same genes. There were 14 genes identified in both the GPA for control traits and the GEA (Table 4). One of these was a ubiquitin‐binding gene. Peptidyl‐prolyl cis‐trans isomerase, involved in protein folding, was known to be heat‐induced in wheat (Kurek et al., 1999). Two genes were involved in glycerophospholipid synthesis or metabolism, suggesting some role related to cell membranes. Aspartyl proteases, like the one linked to winter precipitation and the number of stomatal rows, have been linked to the wood formation and to plant growth and development more generally (Cao et al., 2019). Butanoate–CoA ligases were often involved in the secondary compound synthesis (Beuerle & Pichersky, 2002) and so could be involved in defenses against biotic antagonists or other stress responses. There were 15 genes identified in both the GPA for trait drought responsiveness and the GEA (Table 5). Most share the same functions as those in Table 4. Moreover, two overlapping genes were directly related to the stress response. Gene wsc1 was involved in cell wall biosynthesis under conditions of stress (Maddi et al., 2012; Zu et al., 2001). Gene PAT14 was involved in leaf senescence in response to stresses (Lai et al., 2015; Zeng et al., 2018). However, several of the overlapping genes in each table had unknown functions, and most of these did not match any sequence in the database.

**TABLE 4:** Overlapping genes in GEA and the GPA for traits in control conditions.

Climate variable	Phenotypic variable	Gene name	Gene function
PCK4	NR_AB	MARPO_0050s0076	Ubiquitin binding
PCK4	NR_AB	Unknown	Unknown
PCK4	NR_AB	Gotri_016876	Unknown
PCK4	NR_AB	Peptidyl‐prolyl cis‐trans isomerase	Protein folding, may be heat induced
PCK4	NR_AB	HAD‐superfamily subfamily IIA hydrolase	Glycerophospholipid biosynthesis
PCK4 & TMIN	NR_AB	Unknown	Unknown
PCK4	NR_AB & R2S	Pyridoxal kinase	ATP/ADP conversion
PCK4	R2S	RNA pseudouridine synthase 4, mitochondrial	Synthesis of modified U in RNA (binding, stability)
PCK4	R2S	Unknown	Unknown
PCK4	R2S	Glycerophosphodiester phosphodiesterase	Glycerophospholipid metabolism
PCK4	R2S	MAP3K epsilon protein kinase 1	Control of cell division/expansion
PPTW	NR_AB	Aspartyl protease	Protein breakdown, often involved in plant growth & development
PPTW	R2S	Eukaryotic translation initiation factor 5B‐like	Translation initiation
TMAX	R2S	Butanoate–CoA ligase	Secondary compound metabolism

**TABLE 5:** Overlapping genes in GEA and the GPA for trait drought responsiveness.

Climate variable	Phenotype variable	Gene name	Gene function
PCK4	ΔGR	CSUI_002384	ATP binding
PCK4	ΔGR	LOC109003013	DNA binding; regulation of translation
PCK4	ΔGR	EXO84A	Exocytosis
PCK4	ΔNR_AB	EUGRSUZ_B03992	Oxidoreductase activity
TMAX	ΔNR_AB	L195_g029008	Nucleic acid binding
PCK4	ΔRL	T459_09847	RNA binding
PCK4	ΔRL	AMTR_s00007p00201600	Ubiquitin binding
CWD	ΔRL	NALOC109013111	RNA binding; regulation of translation
PCK4	ΔRL	MARPO_0181s0009	Eoxyribonucleotide catabolic process
PCK4	ΔRL	PAT14	Leaf senescence
PCK4	ΔSD_AD & ΔSW	Unknown	Unknown
PCK4	ΔSW	LOC109001250	Peptidyl‐prolyl cis‐trans isomerase activity
PCK4	ΔSW	wsc1	Regulation of cell wall organization or biogenesis
PCK4	ΔSW	CCAM_LOCUS30844	Unknown
CWD	ΔSW	Unknown	Unknown

## 4 DISCUSSION

In the GEA analysis, over half of the SNPs were associated with April 1st snowpack (PCK4). In this Mediterranean climate region, almost all of the annual precipitation occurs during the winter, and the melting of winter snow accumulation at high elevations feeds spring and summer streamflow (Serreze et al., 1999). Lack of snow can limit seedling establishment (Andrus et al., 2018). A “blanket” of snow can also insulate seedlings from extremely cold temperatures, but may also delay the start of their growing season (Ettinger & HilleRisLambers, 2013; Renard et al., 2016). Consistent with this latter possibility, one of the associated SNPs was in a gene involved in light responses. Winter minimum temperature (TMIN), which has frequently been found to limit growth in tree‐ring studies (Harvey et al., 2020), shows the next highest number of associations. The number of SNPs associated with more than one climatic variable was low (Figure 2), which may indicate that we successfully selected semi‐independent climatic variables that require different genetic adaptations. The highest overlap was between PCK4 and TMIN (64 SNPs) and between CWD and TMIN (17 SNPs). The former SNP set may be related to adaptation to cold and snow depth, while the latter SNP set may be related to how quickly the site warms up in spring, drying out the soil. A similar GEA we conducted for the co‐occurring species *Pinus lambertiana* also identified April snowpack as a key environmental variable that may have shaped local adaptation, and found low overlap in loci associated with different climate variables (Moran et al., 2023).

In the GPA analysis, most SNPs associated with control phenotypic traits were linked with root‐to‐shoot ratio (R2S) and the number of abaxial stomatal rows (NR_AB). In contrast, most SNPs associated with phenotypic responses to drought were linked with shoot weight (SW), root length (RL), and R2S. Drought‐stressed ponderosa pine seedlings allocated more to their root system, with longer root length, higher root‐to‐shoot dry mass ratio, less dry shoot mass, and less height growth. Other studies in pines have found similar patterns (Cregg & Zhang, 2001; Irvine et al., 1998; Seiler & Johnson, 1988; Taeger et al., 2015). This may indicate investment in greater water harvesting capacity at the cost of the overall low growth of aboveground structures – though low shoot growth can have the benefit of further reducing transpirational water loss (Moran et al., 2017). We found that dry treatment root‐to‐shoot ratio was positively associated with survival in that treatment (Wu et al., 2023). Many of the SNPs associated with phenotypic drought responses were in genes associated with cell division & differentiation and with root growth, both of which make sense in light of the observed changes in allocation to root versus shoot growth. The number of SNPs associated with more than one trait was low in both GPA analyses. The highest degree of overlap was in drought responsiveness of RL and R2S and of R2S and NR_AB (Figure 6).

Non‐synonymous (AKA missense) variants that may directly affect phenotype by changing protein form and function included 195 of the climate‐associated, 93 of the control environment phenotype‐associated, and 140 of the phenotype drought‐response‐associated SNPs (Tables 1, 2, 3). Intragenic or synonymous variants are assumed to be neutral with respect to fitness but might be in linkage disequilibrium with a nearby causal variant. While linkage disequilibrium is usually low in conifers (Neale & Savolainen, 2004), the GBS sequence fragments were relatively short (90–100 bp or less) and were trimmed further before SNP calling, so a linked non‐synonymous variant could have been missed. We also found quite a few upstream and downstream SNPs in both GEA and GPA analyses that might directly affect gene expression or be linked to a protein‐altering variant.

While we found no overlaps in specific SNPs between our GEA and GPA, we did identify several SNP‐containing genes that were the same across the analyses (Tables 4 and 5). Most of these genes have been linked to stress responses in other studies. For example, gene wsc1 is involved in cell wall biosynthesis and gene PAT14 is involved in leaf senescence, both in response to stress (Lai et al., 2015; Maddi et al., 2012; Zeng et al., 2018; Zu et al., 2001). Moreover, there was substantial overlap in functional categories found to be directly related to drought tolerance or other environmental responses in previous studies (Figures 3, 4, 5). The prevalence of genetic associations related to abscisic acid (ABA)‐signaling pathways and ubiquitination in GEA and GPA analyses is consistent with prior observations (Moran et al., 2017) and with results of the *P. lambertiana* analysis (Moran et al., 2023). Increasing ABA concentrations are used as a signal to keep stomata closed during dry conditions, reducing water loss (Brodribb et al., 2014). In addition, ABA signaling can also affect shoot growth and water uptake (Buckley, 2005; Hamanishi & Campbell, 2011). Ubiquitination is involved in drought responses in model species by playing a role in ABA‐mediated dehydration stress responses (Kim et al., 2012; Ryu et al., 2010) or through the downregulation of plasma membrane aquaporin levels (Lee et al., 2009). Notably, aquaporin genes, which are crucial for adjusting stomatal conductance under water stress, have been identified in both poplar studies (Secchi & Zwieniecki, 2014) and GEA studies focused on oaks from dry environments (Temunović et al., 2020). Such findings underscore the significance of ubiquitin‐mediated processes in the drought responses of a wide range of tree species. However, our understanding of the role of ubiquitin in conifer drought response is still somewhat limited. A study in black spruce (*Picea mariana*) identified 16 candidate genes correlated with precipitation, including the genes in the ubiquitin protein handling pathway (Prunier et al., 2011). The association between ubiquitin protein and roots and stomatal density may indicate previously unidentified roles in drought response.

Moreover, genes associated with seeds and seed dormancy can also be directly involved in drought tolerance; for instance, dehydrins can protect proteins from desiccation in both seeds and other plant tissues (Moran et al., 2017). However, reproduction‐related genes might also show associations with environmental gradients if they are involved in reproductive timing. Genes involved in xylem & phloem differentiation or cell wall formation could shape the hydraulic safety of water‐transporting cells, which can be quite plastic in pines (Lauder et al., 2019). Other than these functions directly related to drought tolerance or different environmental responses, the other overlapping functions among GEA and GPA analyses are involved in gene expression (RNA or DNA binding, transcription factors, helicase activity, ribosome components, methylation) or ATP binding (motifs found in membrane transporters, microtubule subunits, enzymes, and other cell components that require energy). Our findings suggest the efficiency of combining GEA and GPA analyses with GBS to uncover potentially important adaptive genetic variation.

In conclusion, by investigating adaptive genetic variation in ponderosa pine with GEA and GPA association analysis, our study found thousands of genomic variants associated with response to climate and physiological traits. Some of these have previously identified functions associated with drought responses, but for others, the gene function – or how that function is relevant for environmental responses – is still unknown. Molecular tools based on the associated genetic markers could be developed to assist breeders and land managers speed up selection for drought tolerance or selecting appropriate seed sources for a changing climate. In addition, our results should open new opportunities for functional studies to determine the molecular roles of the genes underlying these associated genetic makers in influencing trees' adaptation.

The two environmental variables with the most genetic associations – snowpack and winter temperatures – are among those that have already undergone significant shifts in recent decades, with further substantial shifts being projected due to anthropogenic climate change (Fyfe et al., 2017; Rapacciuolo et al., 2014). This suggests that tree populations in the Western US will be under rapidly shifting selective pressures, making exploring the potential of genomic selection for seed selection of pressing concern. We found considerable heritable variation in drought‐responsive traits (Wu et al., 2023), suggesting adaptive potential exists if the change is not too rapid. We are also following up on this study by testing the ability of the SNP associations detected here to predict performance in post‐fire restoration plantings.

## AUTHOR CONTRIBUTIONS

**Mengjun Shu:** Data curation (lead); formal analysis (lead); investigation (equal); writing – original draft (lead); writing – review and editing (equal). **Emily V. Moran:** Methodology (equal); writing – review and editing (equal).

## CONFLICT OF INTEREST STATEMENT

The authors declare no conflicts of interest.

## Supporting information


Figures S1–S6

## Data Availability

Raw DNA sequencing data: available at National Center for Biotechnology Information under BioProject number PRJNA707049. https://www.ncbi.nlm.nih.gov/bioproject/PRJNA707049. Individual tree and seedling SNP genotypes and annotation for significantly associated SNPs are available on Dryad. DOI: https://doi.org/10.6071/M3DQ1D. Greenhouse seedling data can be found as a supplement (Wu et al., 2023).
