# Supplementary material for: Identifying genetic variation associated with environmental gradients and drought‐tolerance phenotypes in ponderosa pine
Source: Ecol Evol. 2023 Oct 13;13(10):e10620. doi: 10.1002/ece3.10620 (PMC10576020; doi:10.1002/ece3.10620)

Appendix:


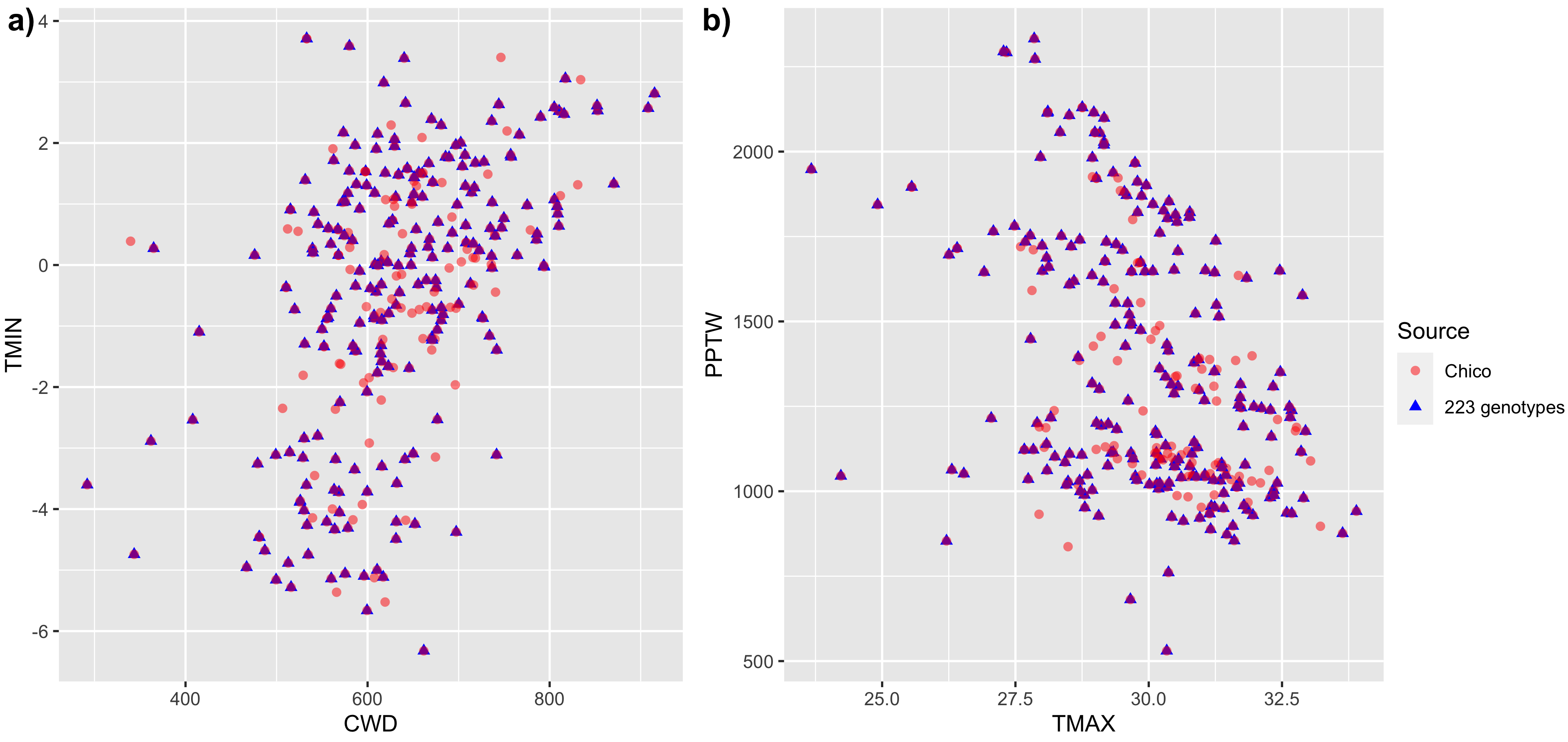
Fig. S1 Comparative analysis of climatic variables between the Chico collection and selected 223 genotypes for association analysis. a) Climatic Water Deficit (CWD) vs. Minimum Winter Temperature (TMIN). b) Maximum Summer Temperature (TMAX) vs. Monthly Winter Precipitation (PPTW).

Fig. S2 PCA analysis of 30-year averages (1921-1950) of all the 18 environmental variables from BCM model.


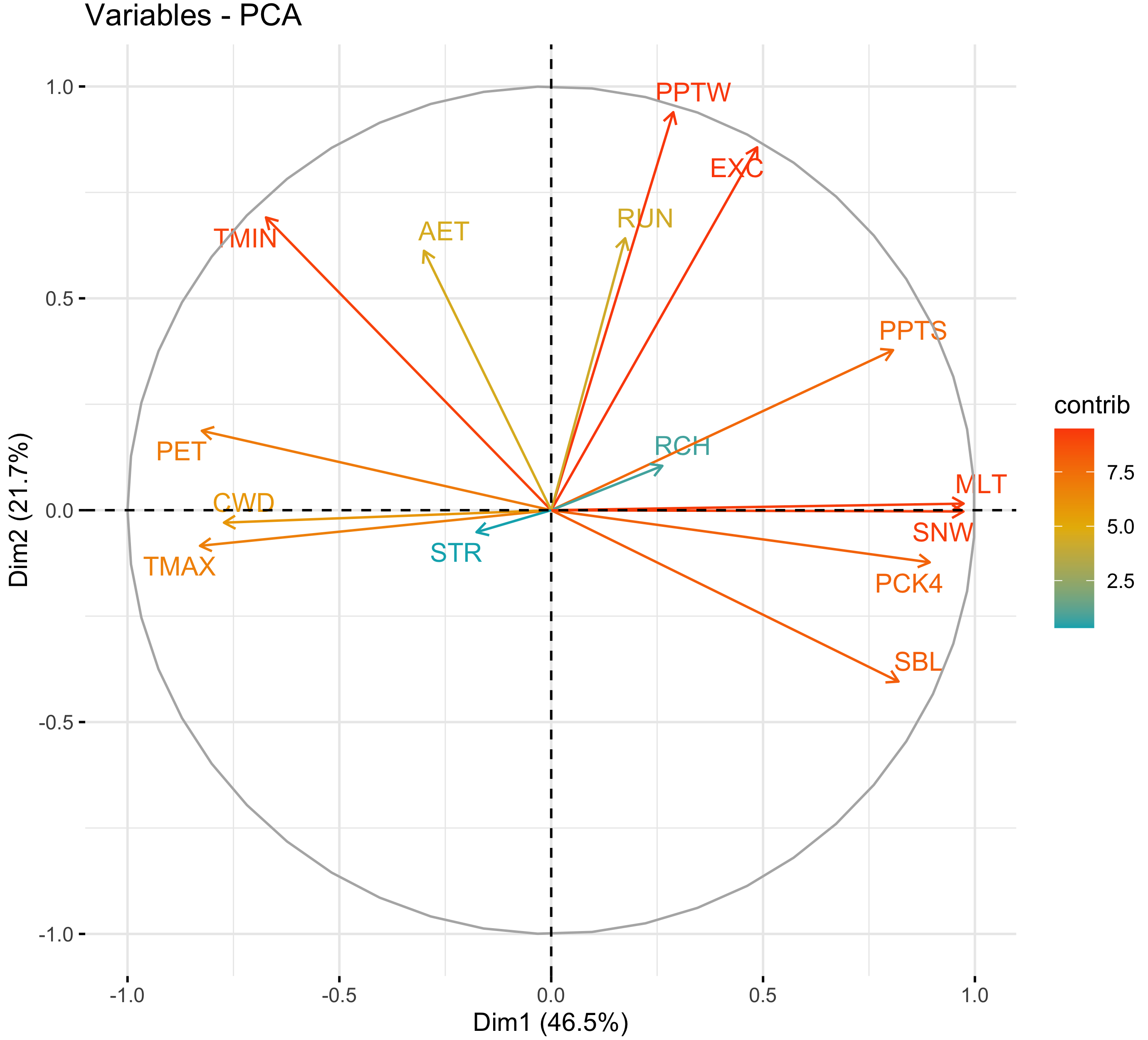


Fig. S3 Comparative analysis of climatic variables between the selected 223 genotypes for association analysis and the 50 mother trees for greenhouse experiment. a) Climatic Water Deficit (CWD) vs. Minimum Winter Temperature (TMIN). b) Maximum Summer Temperature (TMAX) vs. Monthly Winter Precipitation (PPTW).


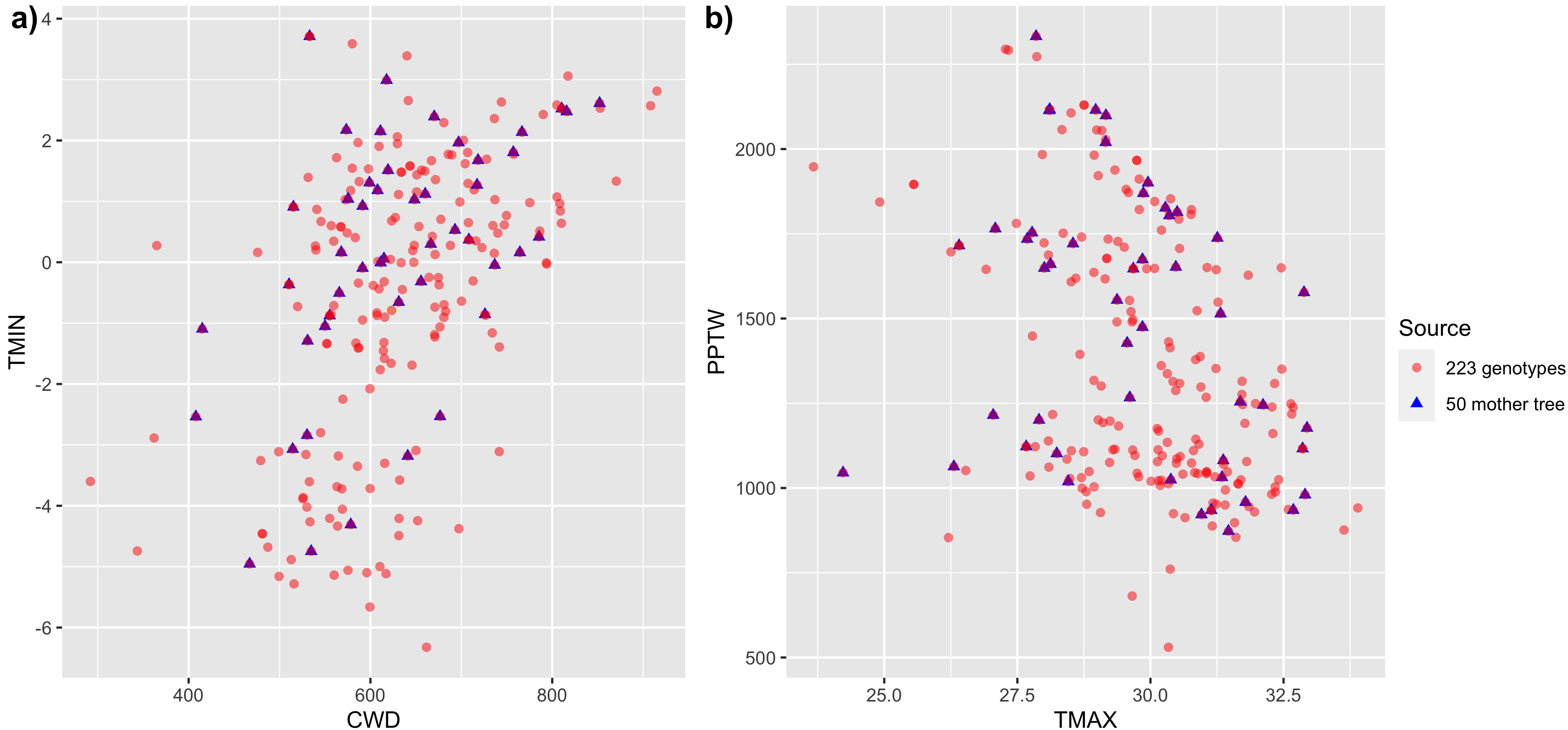


Fig. S4 Principal component analysis (PCA) of 223 Pinus ponderosa individuals using all 4,155,896 SNPs

**
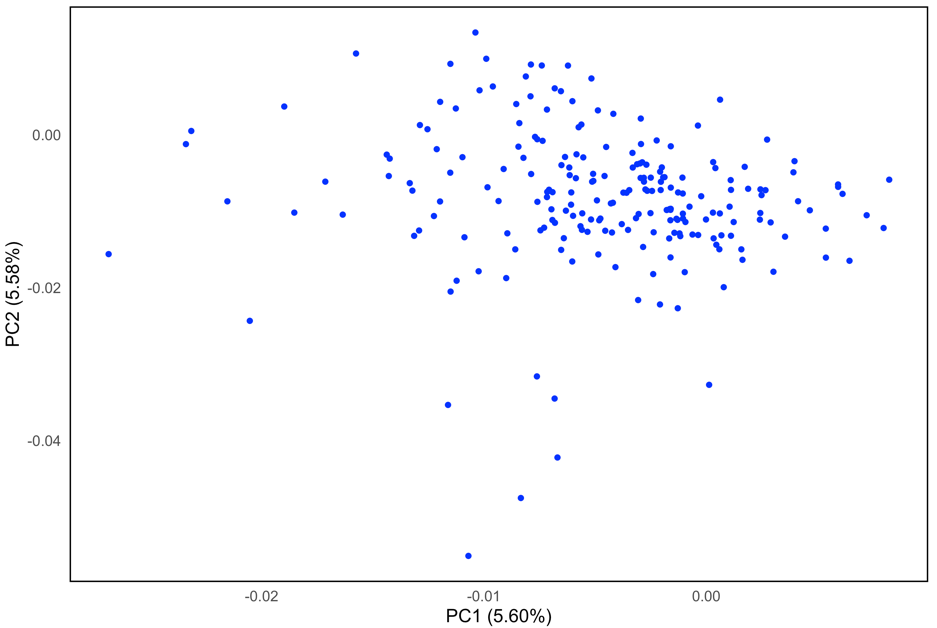
**

Fig. S5 Plot of Cross-validation (CV) error of 223 ponderosa pine individuals based on a total of 4,155,896 SNPs at K=1, 2, 3, 4, 5, 6


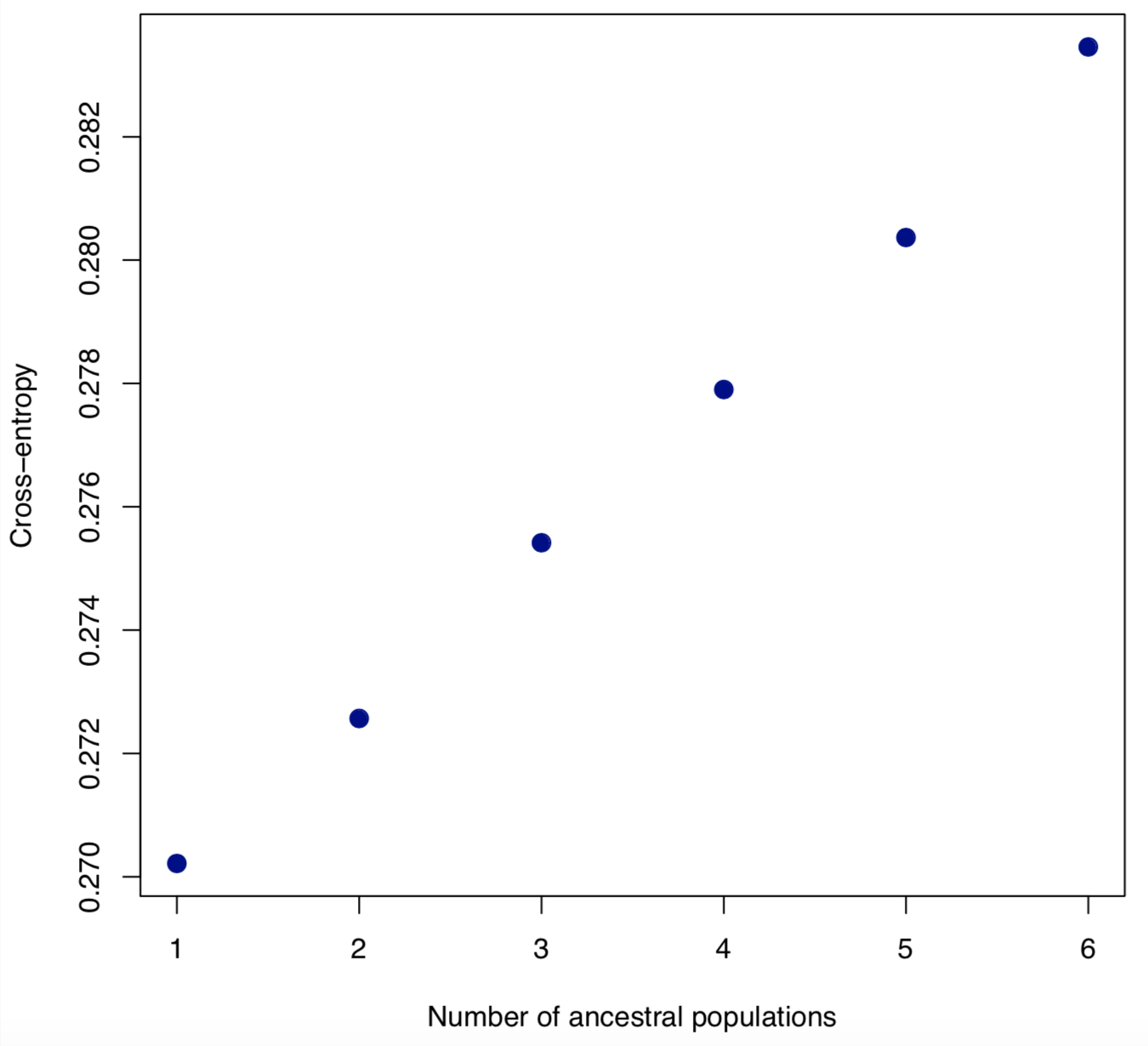


Fig. S6 Admixture analysis of 223 individuals based on a total of 4,155,896 SNPs at K=2.


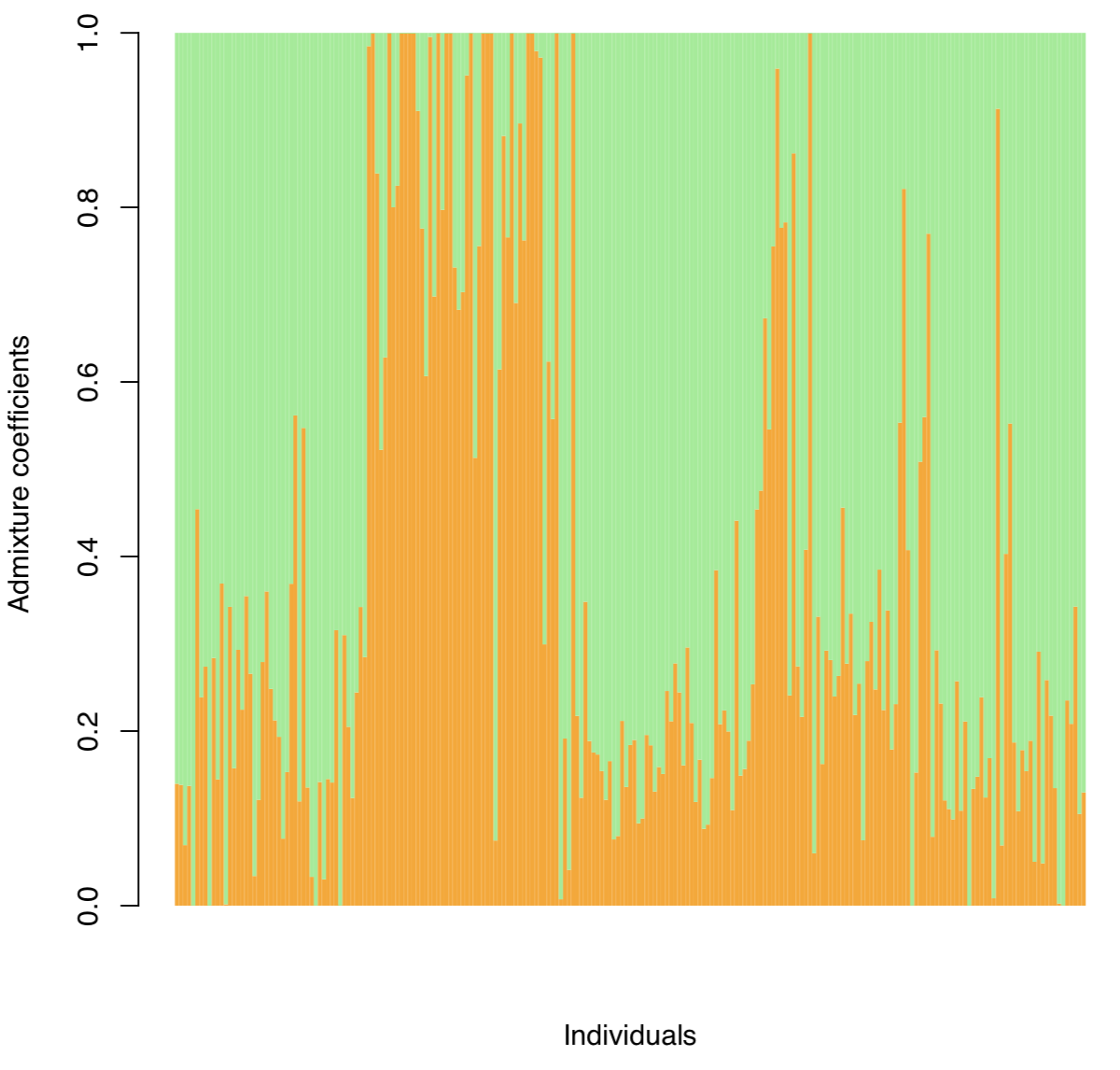

Supplement: Supplementary file 1 — Figures S1–S6 [file ECE3-13-e10620-s001.docx]
